# A Meta-Analysis and Meta-Regression of Frequency and Risk Factors for Poststroke Complex Regional Pain Syndrome

**DOI:** 10.3390/medicina57111232

**Published:** 2021-11-11

**Authors:** Yu-Chi Su, Yao-Hong Guo, Pei-Chun Hsieh, Yu-Ching Lin

**Affiliations:** 1 National Cheng Kung University Hospital, College of Medicine, National Cheng Kung University, Tainan 70428, Taiwan; zac850429@gmail.com; 2 Department of Physical Medicine and Rehabilitation, National Cheng Kung University Hospital, College of Medicine, National Cheng Kung University, Tainan 70428, Taiwan; patchguo@gmail.com (Y.-H.G.); hsieh.pei@msa.hinet.net (P.-C.H.); 3 Department of Physical Medicine and Rehabilitation, College of Medicine, National Cheng Kung University, Tainan 70428, Taiwan

**Keywords:** complex regional pain syndrome, poststroke, risk factors, frequency, incidence, prevalence

## Abstract

*Background and Objectives:* This article aimed to investigate the risk factors for poststroke complex regional pain syndrome (CRPS). *Materials and Methods:* We searched electronic databases including PubMed, Medline, Web of Science, Cochrane Library, and Embase up to 27 October 2021. We enrolled analytical epidemiological studies comprising cohort, case-control, and cross-sectional studies. A quality assessment was performed using the Newcastle–Ottawa Quality Assessment Scale for cohort and case-control studies and the Joanna Briggs Institute critical appraisal checklist for analytical cross-sectional studies. Binary outcomes were reported as odds ratios (ORs), and continuous outcomes were described as standardized mean differences (SMDs) with 95% confidence intervals. For the meta-regression, beta coefficient and *p* value were adopted. *Results:* We included 21 articles comprising 2225 participants. Individuals with shoulder subluxation and spasticity were found to have higher risks for poststroke CRPS. Spasticity with higher modified Ashworth scale score, lower Brunnstrom hand stage, and inferior Barthel index scores were observed in patients with poststroke CRPS. The pooled incidence proportion in nine articles was 31.7%, and a correlation was found between effect sizes and the ratio of women and the proportion of left hemiparesis. The summarized prevalence in nine cross-sectional studies was 33.1%, and a correlation was observed between prevalence and the subluxation ratio and Brunnstrom stage. *Conclusions:* Based on our meta-analysis, being female, left hemiparesis, shoulder subluxation, spasticity, a lower Brunnstrom stage of distal upper limb, and an inferior Barthel index are all features for poststroke CRPS. Larger studies with greater statistical power may confirm our findings and clarify some other unknown risk factors for poststroke CRPS.

## 1. Introduction

Complex regional pain syndrome (CRPS) is a clinical syndrome characterized by pain, sensory, motor, vasomotor, and trophic changes [1]. Different diagnostic criteria have been proposed, including the Budapest criteria, which is currently the criteria most commonly adopted [2]. Most poststroke CRPS is considered CRPS type one, also known as reflex sympathetic dystrophy. The pathogenesis of poststroke CRPS is still unclear [3], and the prevalence varies from 2% to 50% based on previous studies [4]. Poststroke CRPS usually occurs one to six months after a cerebrovascular accident (CVA), which happens to be the period with the highest potential for rehabilitation [5]. Hence, prevention, early diagnosis, and treatment of poststroke CRPS are important following a stroke.

Currently, no guidelines for the prevention of poststroke CRPS have been established. Some investigations have revealed early rehabilitation may decrease the likelihood of CRPS [6,7]. Clinical and radiological features for poststroke CRPS have been proposed, such as glenohumeral subluxation, severity of motor deficits, immobilization of upper extremity, and involvement of corona radiate [8]. Geurts et al. [9] conducted a systematic review of the etiology of shoulder-hand syndrome in 2000. However, the study did not include a quantitative synthesis, and many articles have been published since then with varying results. Furthermore, identifying risk factors for poststroke CRPS may make it possible to recognize the subgroup of patients who need more attention and preventive interventions.

This article of systematic review and meta-regression was aimed toward comprehensively summarizing the epidemiological literature on poststroke CRPS. We included both prospective and retrospective studies.

## 2. Materials and Methods

This systematic review and meta-analysis followed the Meta-analysis of Observational Studies in Epidemiology (MOOSE) reporting guidelines [10]. We did not register or publish a prior protocol.

### 2.1. Eligibility Criteria

All analytical epidemiological studies that provided risk factors for poststroke CRPS were included. The target population was patients who had experienced cerebrovascular accidents, regardless of the type of stroke, age, sex, the severity of the neurological sequelae, or the etiology. We did not set restrictions for the type of exposure, and the diagnostic criteria for poststroke CRPS were not limited. The publication language was restricted to English.

We excluded articles specifically assessing bone scintigraphy as a determinant since they have been recently surveyed [11,12,13]. We omitted papers targeting risk factors within a general population sample in which patients with poststroke CRPS were compared with non-stroke controls. This is because it is impossible to distinguish whether the determinants identified in these studies were simply predictive of stroke, or whether they were specific to poststroke CRPS. Investigations restricted to clinical subgroups with specific comorbidities or complications, such as diabetes mellitus or shoulder joint subluxation, were also excluded, since their risk factors for poststroke CRPS may differ. Case reports and conference proceedings were omitted due to a high risk of publication bias.

### 2.2. Search Strategy

PubMed, Cochrane Central Register of Controlled Trials, Web of Science, Medline, and Embase were searched with the language restricted to English. Keywords included “stroke” AND “complex regional pain syndrome.” Search time was from inception to the present time. The final search was carried out on 27 October 2021 (see File S1 in Supplementary Materials. for the full search strategy).

### 2.3. Study Selection and Data Extraction

Two authors (Y.-C.S. and Y.-H.G.) reviewed the titles and abstracts considered for study inclusion, and the full texts were then retrieved for assessment. Reference lists of all retrieved works were searched to identify further related papers. To assess the reliability of the eligibility criteria, another author (P.-C.H.) reviewed a random sample of all 10% of all of the considered articles. A kappa statistic for the degree of agreement between reviewers was then calculated, which was as high as 0.77 [14]. The senior author (Y.-C.L.) made the final decision if a consensus could not be made between the reviewers. We used a data extraction sheet for data collection, which included first author, year of publication, study design, patient demographics, risk factors, and proportion of patients diagnosed with poststroke CRPS. If median and interquartile range were reported rather than mean and standard deviation, we used the quantile estimation approach proposed by McGrath et al. [15]. As for data presented as charts, NIH (National Institutes of Health) Image was adopted [16]. The authors were contacted as necessary to resolve any uncertainties.

### 2.4. Quality Assessment

We used the Newcastle–Ottawa Quality Assessment Scale for the cohort and case-control studies and the Joanna Briggs Institute (JBI) critical appraisal checklist for analytical cross-sectional studies [17]. Two authors (Y.-C.S. and Y.-H.G.) independently evaluated the articles, and disagreements were resolved by discussion with the senior author (Y.-C.L.). Reviewer Manager version 5.3 was utilized to summarize the results in the form of a graph of risk of bias and summary table.

### 2.5. Statistical Analysis

The primary outcomes were factors considered to contribute to the frequency of poststroke CRPS. We conducted a meta-analysis if a determinant was appropriately mentioned at least three times in similar populations. The odds ratios (ORs) with 95% confidence intervals (CI) were reported for binary outcomes, and standardized mean differences (SMDs) with a 95% CI were used for the continuous outcomes. The secondary outcome was the frequency of CRPS following a CVA, presented with a 95% CI. We used a random effects model for the pooling of effect sizes. Between-study heterogeneity was assessed using I^2^. Moderate heterogeneity was defined as an I^2^ > 50% and high heterogeneity as an I^2^ > 75% [18]. Post hoc analyses were conducted for outcomes with I^2^ > 50%. This included random effects meta-regression that explored the correlations between effect sizes and the different characteristics of the study populations. Continuous variables comprised age, proportion of females, rate of hemorrhagic stroke, ratio of left hemiparesis, duration of the CVA, proportion of subluxation, and the Brunnstrom stage. If an article reported the Brunnstrom stage of both arm and hand, the mean of the two was adopted for post hoc analysis. The categorical variable was the diagnostic criteria for CRPS. The results of the meta-regression were considered to be statistically significant when *p* < 0.05. Funnel plots and Egger’s test were used to assess publication bias, and a two tailed *p* < 0.1 was regarded as statistically significant [19]. Sensitivity analyses were also carried out by removing cross-sectional studies to determine their contribution to the overall effect size in the meta-analyses of primary outcomes. The statistical analyses were conducted using Comprehensive Meta-analysis Software version 3 (Biostat, Englewood, NJ, USA).

## 3. Results

### 3.1. Study Selection and Description

**Figure 1:**
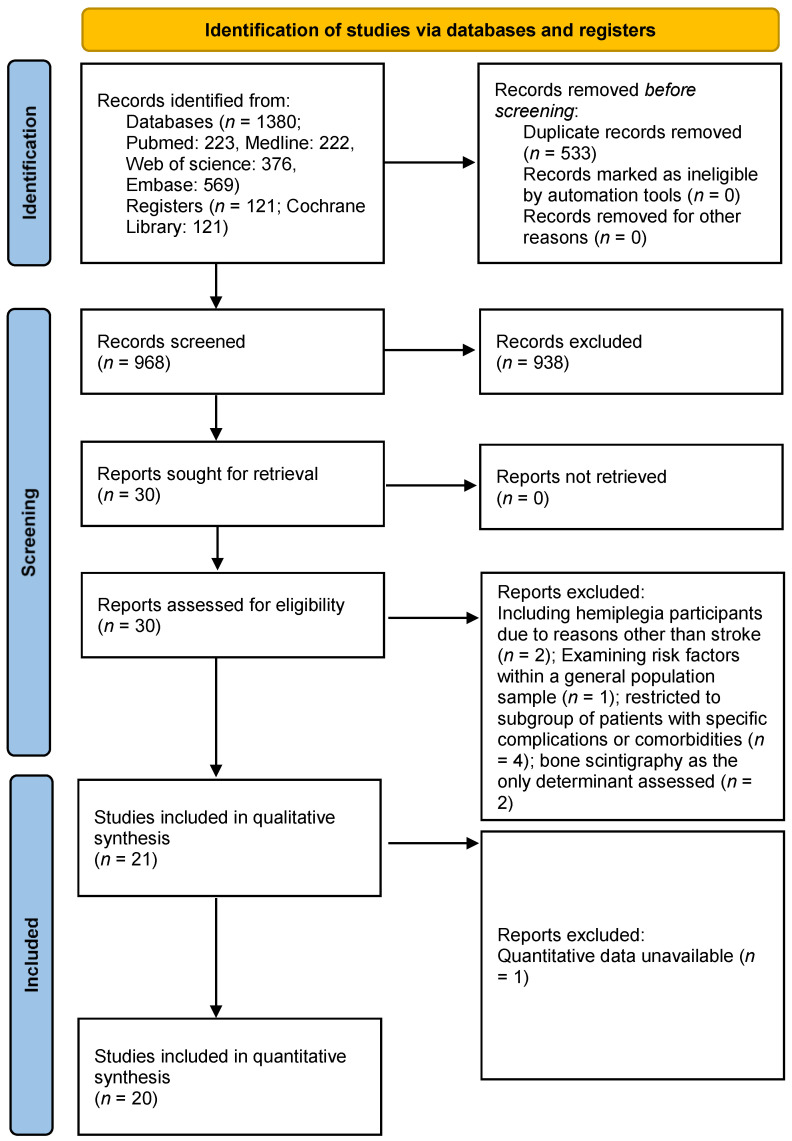
Literature screening process and results.

The initial search showed 1501 articles. Twenty-one studies [4,8,20,21,22,23,24,25,26,27,28,29,30,31,32,33,34,35,36,37,38] underwent a qualitative synthesis (Figure 1). The main features of the included trials are summarized in Table 1. Details of the articles excluded after full text retrieved are presented in File S3 in Supplementary Materials.

Three cohort studies [31,32,37], eight nested case-control studies [8,20,23,24,26,30,35,36], and ten cross-sectional studies [4,21,22,25,27,28,29,33,34,38] were enrolled. The number of participants ranged from 20 to 426 subjects. In eighteen papers [4,8,20,21,23,24,25,26,28,29,30,31,32,33,35,36,37,38], hospitalized patients were recruited, one [22] investigation enrolled subjects from outpatient clinics, and two researchers [27,34] did not specify whether they recruited participants from inpatient or outpatient departments. Various diagnostic criteria were adopted, including clinical and radiological findings. The percentage of patients developing CRPS after experiencing a stroke ranged from 8.3% to 59.4%. Mean ages were available in 17 articles, where the subjects ranged in age from 49 to 67, and one paper reported a median age of 72 (Table 2).

### 3.2. Risk of Bias Assessment

**Figure 2:**
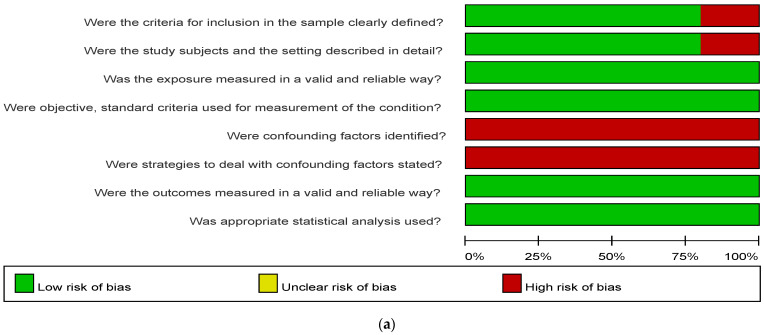

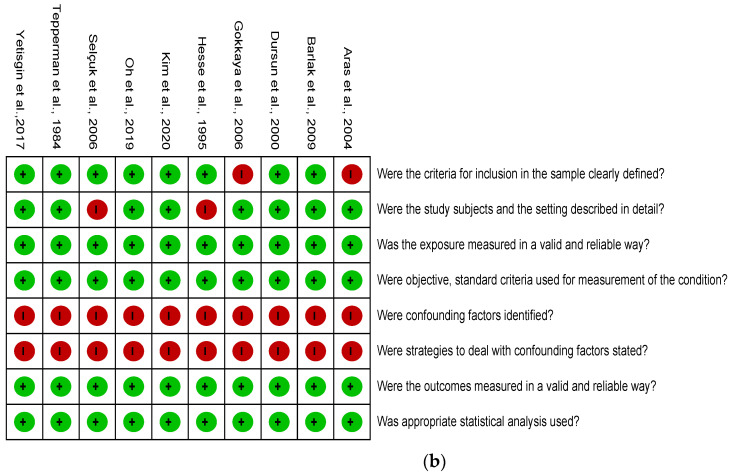
Risk of bias assessment by the Joanna Briggs Institute (JBI) critical appraisal checklist for analytical cross-sectional studies (**a**) Risk of bias graph of cross-sectional studies; (**b**) Risk of bias summary of cross-sectional studies.

Two of the three cohort studies did not demonstrate whether the outcome of interest was presented at the start of the research. One of the eight nested case-control studies did not mention recruitment of all eligible cases within a defined period of time. None of the 11 articles referenced above reported the methods used to increase comparability (Table 3). Two cross-sectional studies did not describe inclusion and exclusion criteria clearly, and another two did not disclose the study setting in detail. Furthermore, none of the 10 cross-sectional studies identified or dealt with confounding factors (Figure 2).

### 3.3. Outcomes

#### 3.3.1. Shoulder Subluxation

**Figure 3:**
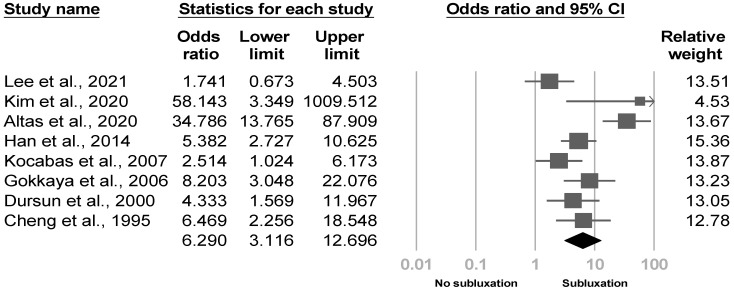
Forest plot of odds ratios in glenohumeral subluxation.

Shoulder subluxation was mentioned in 13 articles [4,8,20,21,23,25,26,28,29,31,33,35,36], and 10 articles [4,20,23,26,28,29,31,33,35,36] revealed significant associations. Pooled effect size of eight [4,8,20,23,26,28,33,35] available investigations showed a higher risk of poststroke CRPS with moderate heterogeneity in patients with glenohumeral subluxation (OR, 6.29, 95% CI, 3.116 to 12.696, I^2^ = 74.3%, Figure 3). The funnel plot and Egger’s test demonstrated no publication bias (*p* = 0.49). Sensitivity analysis did not change the results (OR, 5.548, 95% CI, 2.104 to 14.629). The post hoc analyses found no correlations between effect sizes and the diagnostic criteria (*p* = 0.92), age (*p* = 0.79), proportion of females (*p* = 0.39), rate of hemorrhagic stroke (*p* = 0.26), ratio of left hemiparesis (*p* = 0.48), duration of CVA (*p* = 0.97), proportion of subluxation (*p* = 0.85), and Brunnstrom stage (*p* = 0.36).

#### 3.3.2. Spasticity

**Figure 4:**
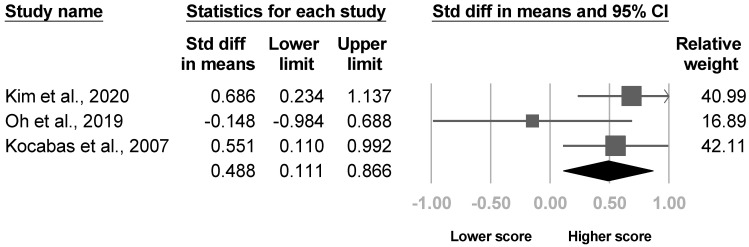
Forest plot of standardized mean differences in Modified Ashworth Scale score.

**Figure 5:**
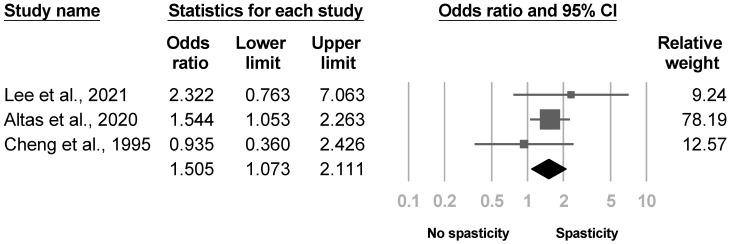
Forest plot of odds ratio in participants with spasticity.

Eight investigations assessed spasticity. Four studies [4,21,26,31] measured the severity of spasticity in patients with and without poststroke CRPS, while the other four [8,20,35,36] compared the proportion of participants with signs of spasticity between groups. Five of the eight articles [4,20,26,31,36] reached statistical significance. Three studies [4,21,26] reporting the severity of spasticity were eligible for the meta-analysis, where the results indicated more severe spasticity in patients with poststroke CRPS with low heterogeneity (SMD, 0.488, 95% CI, 0.111 to 0.866, I^2^ = 33.1%, Figure 4). The funnel plot and Egger’s test did not reveal publication bias (*p* = 0.18). Sensitivity analysis was not performed, since only one article was left after excluding cross-sectional studies [26]. Another three investigations [8,20,35] reporting the proportion of patients with spasticity were deemed suitable for the meta-analysis. The results demonstrated a higher risk of poststroke CRPS with low heterogeneity in patients with spasticity (OR, 1.505, 95% CI, 1.073 to 2.111, I^2^ = 0.0%, Figure 5). No publication bias was observed based on the funnel plot and Egger’s test (*p* = 0.94). None of the papers included were cross-sectional studies, so sensitivity analysis was not conducted.

#### 3.3.3. Brunnstrom Stage

**Figure 6:**
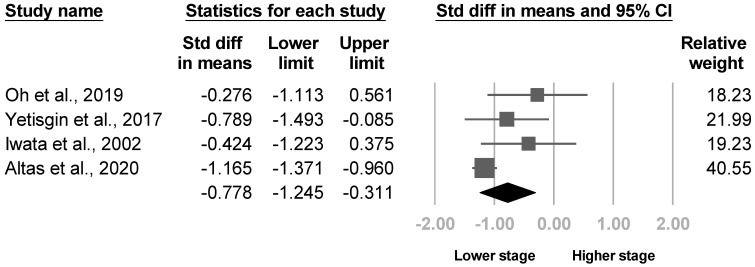
Forest plot of standardized mean differences in the Brunnstrom hand stage.

**Figure 7:**
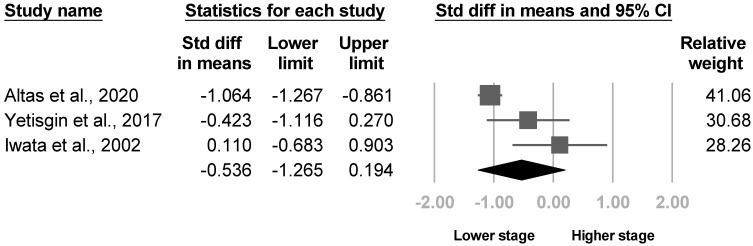
Forest plot of standardized mean differences in the Brunnstrom arm stage.

Eight articles mentioned the Brunnstrom stage. Three of the eight [20,22,30] reported the Brunnstrom stage for the arm and hand separately, and one analyzed hand only [21]. The Brunnstrom hand stages were significant in two of the four studies [20,22], and the Brunnstrom arm stage was significant in one article [20]. The other four investigations did not specify the site of the Brunnstrom stage [25,26,28,33]. Four articles [20,21,22,30] underwent the meta-analysis for the Brunnstrom hand and arm stage, where a lower hand stage was found in patients with poststroke CRPS with significant heterogeneity (SMD, −0.778, 95% CI, −1.245 to −0.311, I^2^ = 59.4%, Figure 6). The funnel plot and Egger’s test indicated significant publication bias (*p* = 0.03, File S2). After removing two cross-sectional studies [21,22], sensitivity analysis remained significant results (SMD, −0.899, 95% CI, −1.596 to −0.203). Nonetheless, no difference in the Brunnstrom arm stage was revealed (SMD, −0.536, 95% CI, −1.265 to 0.194, I2 = 80.6%, Figure 7). No publication bias was found by funnel plot and Egger’s test (*p* = 0.13). Sensitivity analysis remained nonsignificant results after removing one cross-sectional study [22] (SMD, −0.542, 95% CI, −1.686 to 0.602). Although the heterogeneity between studies were high for both Brunnstrom arm and hand stage, post hoc analysis was not conducted due to insufficient article number.

#### 3.3.4. The Barthel Index

**Figure 8:**
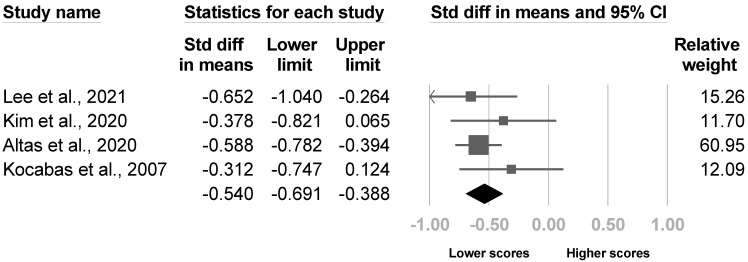
Forest plot of standardized mean differences in the Barthel index.

Four studies evaluated the Barthel index [4,8,20,26], and two studies [8,20] revealed significant between-group differences. The meta-analysis comprised all four papers, and the results indicated lower Barthel indexes in patients with poststroke CRPS with low heterogeneity (SMD, −0.540, 95% CI, −0.691 to −0.388, I^2^ = 0.0%, Figure 8). No publication bias was detected based on the funnel plot and Egger’s test (*p* = 0.35). One article [4] was excluded in sensitivity analysis, and the pooled effect size did not change (SMD, −0.561, 95% CI, −0.772 to −0.4).

#### 3.3.5. Shoulder Pain

**Figure 9:**
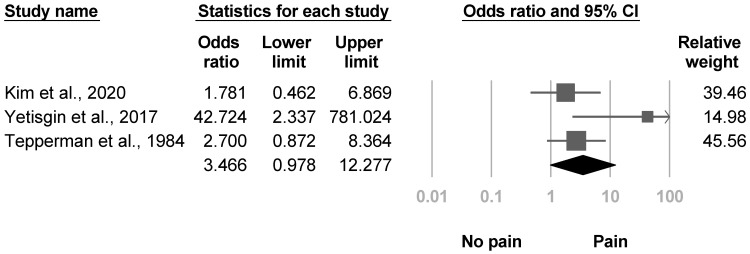
Forest plot of odds ratios in participants with shoulder pain.

Three investigations [4,22,38] reported the proportion of individuals with shoulder pain, and one study described more shoulder pain in patients with poststroke CRPS. The pooled effect sizes were statistically nonsignificant with low heterogeneity (OR, 3.466, 95% CI, 0.978 to 12.277, I^2^ = 47.5%, Figure 9). The funnel plot and Egger’s test did not indicate publication bias (*p* = 0.29). Sensitivity analysis for shoulder pain was not tested because all three articles were cross-sectional studies.

#### 3.3.6. Age

**Figure 10:**
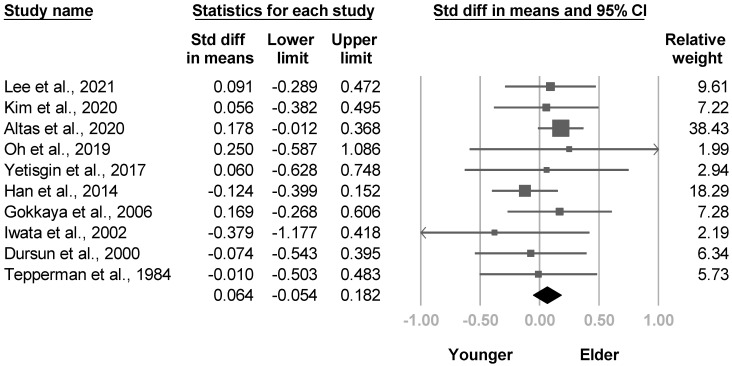
Forest plot of standardized mean differences in age.

Thirteen investigations assessed the effects of age, and none of them showed an association [4,8,20,21,22,23,28,30,31,33,35,36,38]. Ten papers [4,8,20,21,22,23,28,30,33,38] were available for the meta-analysis, and the pooled effect size was not significant with low heterogeneity (SMD, 0.064, 95% CI, −0.054 to 0.182, I^2^ = 0.0%, Figure 10). No publication bias was detected based on the funnel plot and Egger’s test (*p* = 0.31). After removing cross sectional studies, four articles [8,20,23,30] underwent sensitivity analysis, which revealed no change in the results (SMD, 0.040, 95% CI, −0.153 to 0.232).

#### 3.3.7. Sex

**Figure 11:**
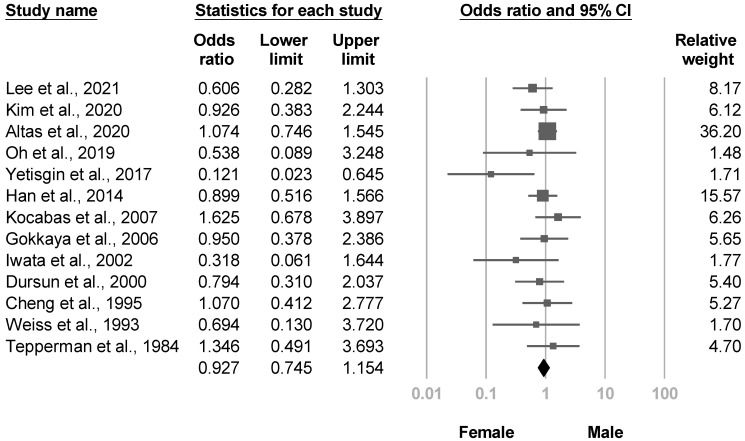
Forest plot of odds ratios for sex.

Fifteen investigations evaluated the effects of sex [4,8,20,21,22,23,26,28,30,31,33,35,36,37,38]. One study [22] reported that female patients had a higher risk of poststroke CRPS. Thirteen studies [4,8,20,21,22,23,26,28,30,33,35,37,38] underwent the meta-analysis, and pooled size effects were not significant with low heterogeneity (OR, 0.927, 95% CI, 0.745 to 1.154, I^2^ = 0.0%, Figure 11). Significant publication bias was found based on the funnel plot and Egger’s test (*p* = 0.06, File S2). After removing six cross-sectional studies, pooled effect size of the remaining articles [8,20,23,26,30,35,37] remained not significant in sensitivity analysis (OR, 0.968, 95% CI, 0.752 to 1.247).

#### 3.3.8. Etiology of Stroke

**Figure 12:**
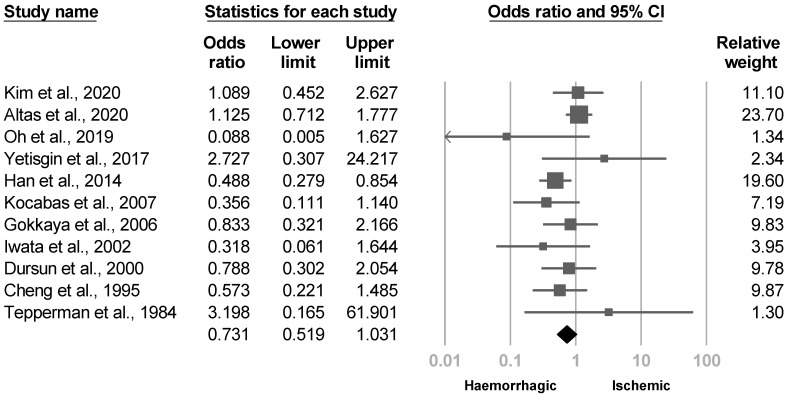
Forest plot of odds ratios for the etiology of stroke.

The etiology of stroke was reported in 12 papers [4,20,21,22,23,26,28,30,33,35,36,38]. Two papers [21,23] showed higher risk of poststroke CRPS in hemorrhagic stroke. Eleven investigations [4,20,21,22,23,26,28,30,33,35,38] were eligible for the meta-analysis, and the pooled effected size was not statistically significant with low between-study heterogeneity (OR, 0.731, 95% CI, 0.519 to 1.031, I^2^ = 24.7%, Figure 12). No publication biases were recognized based on the funnel plot and Egger’s test (*p* = 0.57). Five articles [20,23,26,30,35] underwent sensitivity analysis after six cross-sectional studies were removed, which did not make a difference in the outcome (OR, 0.609, 95% CI, 0.365 to 1.018).

#### 3.3.9. Side

**Figure 13:**
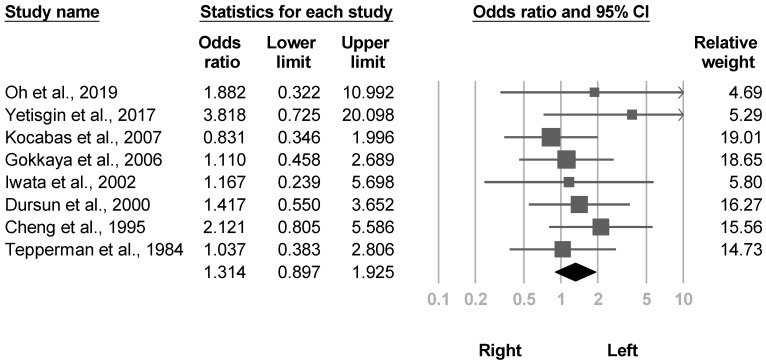
Forest plot of odds ratios for paralytic side.

Fourteen investigations [4,20,21,22,23,26,28,30,31,33,35,36,37,38] reported the effects of lesions on different sides of brain or sides of paralysis. None of them showed statistical significance. Eight studies [21,22,26,28,30,33,35,38] reporting paralytic sides were available for the meta-analysis, and the pooled effect size was nonsignificant with low heterogeneity (OR, 1.314, 95% CI, 0.897 to 1.925, I^2^ = 0.0%, Figure 13). No publication bias was detected based on funnel plot and Egger’s test (*p* = 0.17). Three articles [26,30,35] were included in the sensitivity analysis, which revealed similar results (OR, 1.252, 95% CI, 0.686 to 2.284).

**Figure 14:**
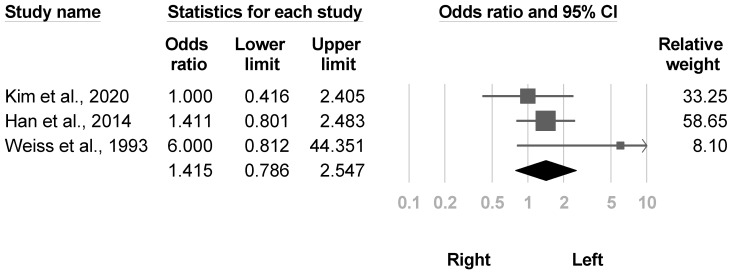
Forest plot of odds ratios for lesion side.

Three studies [4,23,37] that mentioned the sides of brain lesions were eligible for the meta-analysis, and the between-group results were nonsignificant (OR, 1.415, 95% CI, 0.786 to 2.547, I^2^ = 22.9%, Figure 14). The funnel plot and Egger’s test did not indicate publication bias (*p* = 0.54). Sensitivity analysis addressed no difference after one cross-sectional study [4] was removed (OR, 2.089, 95% CI, 0.592 to 7.379).

#### 3.3.10. Duration of Stroke

**Figure 15:**
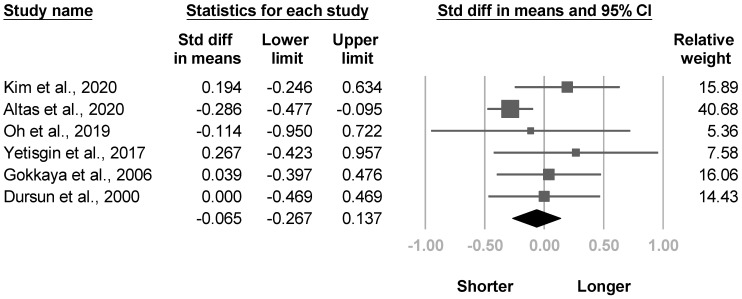
Forest plot of standardized mean differences in duration of stroke.

Six articles [4,20,21,22,28,33] compared the duration of the CVA between individuals with and without CRPS. One article [20] exhibited a shorter stroke duration in patients with poststroke CRPS. The meta-analysis included all six studies, and no between-group differences were found with low heterogeneity (SMD, −0.065, 95% CI, −0.267 to 0.137, I^2^ = 26.3%, Figure 15). Significant publication bias was found based on the funnel plot and Egger’s test (*p* = 0.05, File S2). Sensitivity analysis for duration of stroke was not conducted because only one [20] article was left after removing the cross-sectional studies.

#### 3.3.11. Frequency

**Figure 16:**
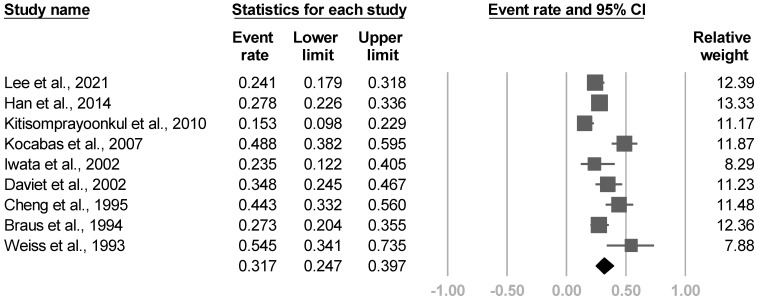
Forest plot of incidence proportion of poststroke complex regional pain syndrome.

Eighteen investigations [4,8,21,22,23,24,25,26,27,28,29,30,31,33,35,36,37,38] outlined the disease frequency. Nine [8,23,24,26,30,31,35,36,37] reported the incidence proportion of poststroke CRPS, and the other nine [4,21,22,25,27,28,29,33,38] mentioned the prevalence. The pooled incidence proportion of nine articles was 31.7% (95% CI, 24.7% to 39.7%, I^2^ = 80.8%, Figure 16). The funnel plot and Egger’s test revealed no publication bias (*p* = 0.52). The post hoc analysis showed significant correlations between effect sizes, the proportion of females (β = 6.1719, *p* = 0.016, File S2), and proportion of left hemiparesis (β = 10.4619, *p* = 0.019, File S2). However, no relationship was found between the incidence proportion and age (*p* = 0.75), proportion of hemorrhagic stroke (*p* = 0.87), and subluxation (*p* = 0.69). Distinct diagnostic criteria, duration of stroke, and the Brunnstrom stage were not assessed due to the small number of studies.

**Figure 17:**
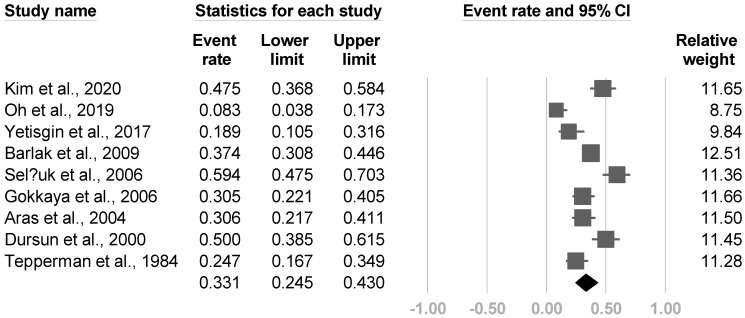
Forest plot of prevalence of poststroke complex regional pain syndrome.

In terms of prevalence, the summarized effect size of nine articles was 33.1% (95% CI, 24.5% to 43%, I2 = 86.0%, Figure 17). No publication bias was identified based on the funnel plot and Egger’s test (*p* = 0.18). The post hoc analysis demonstrated a significant correlation between the prevalence and the proportion of shoulder subluxations (β = 1.9162, *p* = 0.011) and the Brunnstrom stage (β = −2.2548, *p* < 0.0001). Nonetheless, the diagnostic criteria (*p* = 0.73), age (*p* = 0.08), sex (*p* = 0.06), proportion of hemorrhagic stroke (*p* = 0.87), side of paralysis (*p* = 0.12), and duration of stroke (*p* = 0.16) did not reach statistical significance.

#### 3.3.12. Other Risk Factors

Three studies [20,30,31] assessed visual neglect, and one study [20] reported statistical significance. Four investigations [26,31,35,37] evaluated sensory impairment, and one [35] showed positive results. Two articles [24,26] calculated the range of motion (ROM) of the shoulder joint, and both were statistically significant. Six studies [4,8,21,26,31,36] measured the strength of the shoulder or wrist, and five [4,8,26,31,36] demonstrated association with poststroke CRPS. Two [4,8] carried out the Mini-Mental State Examination (MMSE), and one study [8] found a significant correlation. Four [4,20,26,31] documented depression with distinct measurements, and two [20,26] reached statistical significance. Two articles [8,23] found a connection between somatosensory evoked potentials (SSEPs) of the median nerve and poststroke CRPS, while another two [27,34] found a linkage with the amplitude of the sympathetic skin response (SSR). One [20] of the two [20,28] investigations reporting duration of hospitalization was statistically significant. Two studies [4,8] reported a higher risk in patients with brain lesions damaging the corticospinal tract although no similar relationships were found in the volume of brain lesions. No increased risk was observed in individuals with diabetes mellitus [20,31,38], hypertension [20,31,38], and atrial fibrillation [20,38].

## 4. Discussion

Our systematic review and meta-analysis identified 21 analytical epidemiological studies investigating risk factors for poststroke CRPS. The results of the meta-analysis demonstrated risk factors, including shoulder subluxation, spasticity, lower Brunnstrom hand stage, and an inferior Barthel index. The post hoc analysis revealed a positive correlation between the incidence proportion with women and left hemiparesis. In addition, a positive correlation between the prevalence with shoulder subluxation, and a negative correlation between prevalence with the Brunnstrom stage were observed in the post hoc analysis. Age, side of lesion, etiology of the stroke, the Brunnstrom arm stage, the duration of a CVA, and shoulder pain were not found to be associated with poststroke CRPS.

Although the pathophysiology of poststroke CRPS remained unclear, some scholars suggested that repeated microtraumas in the shoulder joint caused chronic pain and the initiation of an abnormal sensory-sympathetic reflex arch [39]. In stroke patients, the stability of the glenohumeral joint may be affected due to paresis or palsy of the shoulder girdle muscles. Such instability may further cause injury in the shoulder joint [20]. In addition, spasticity of shoulder muscles may cause glenohumeral capsulitis and pain, and some researchers suggested that it contributes to CRPS [40]. These hypotheses correlated with the conclusions of our review. Furthermore, the occurrence of shoulder subluxation has been reported to be negatively correlated with the Brunnstrom stage [41], which may explain the findings in our work. As for Barthel index, the effect observed in our review may have been derived from the positive correlation between the index and the Brunnstrom stage [42].

Daviet et al. [31] concluded that shoulder subluxation may only play a secondary role in the development of poststroke CRPS, being a reflection of the severity of paresis. However, a later published article by Kocabas et al. [26] disagreed such statement and further concluded that subluxation can also be a factor for the development of type 1 CRPS. We believe that the relationship between subluxation and poststroke CRPS needs further investigation given that there was significance between study heterogeneity detected in our review. Moreover, our summarized odds ratio was derived from studies that were not matched for severity of paresis, meaning that we could not support nor oppose the conclusion of Daviet et al. Future studies that are matched for severity of paresis are warranted to delineate the relationship between shoulder subluxation and poststroke CRPS.

In the post hoc analysis, women and paralysis of left limbs were found to be more likely to be associated with poststroke CRPS. In a population-based study [43], female patients had a higher incidence of CRPS compared with male patients, which may explain the finding in our review, although the mechanisms are not clear so far. Furthermore, one article showed that patients with hemianopia or hemineglect were more subject to CRPS [20]. Since both hemineglect and weakness left side limbs occur more often in a right hemispheric stroke, we hypothesized that patients with right hemispheric lesions have a higher risk for traumatizing the contralateral shoulder due to neglect syndromes [36,44].

There were several limitations in this article. First, various diagnostic criteria were adopted. Variations in criteria may derive distinct risk factors [21] and may also decrease the generalizability of our results. A full assessment of contributing elements using distinct diagnostic criteria was not possible here due to low numbers of studies. In recent years, the Budapest criteria has become the mainstream diagnostic tool for CRPS, and larger cohorts based on such criteria are warranted to confirm the findings of our review. Second, we could not conduct a meta-analysis for neglect because of too few researchers, and the summarized effect of side of brain lesions in radiological findings were non-significant probably due to insufficient statistical power. To form a stronger link between left hemiparesis and poststroke CRPS, delineating these two factors of neglect and side of brain lesions is necessary in further research. Third, research that failed to achieve statistical significance may have gone unpublished, which may have caused false positives. Brunnstrom stage of the hand may possess such publication bias in our meta-analysis, and future research was necessary to delineate the influence of such bias. Fourth, only a few of the enrolled articles adopted adjustments for confounding factors. Hence, we could not conduct a sensitivity analysis to estimate the effects of confounding factors on our results. Fifth, most of the study population in our review was recruited from among hospitalized patients. Investigations targeting individuals from outpatient departments are needed to clarify the disease frequency and contributing factors in such groups. Finally, non-English publications were not enrolled in our review; nonetheless, the authors believe that it was unlikely to cause the exclusion of any major articles.

This review highlighted several risk factors of poststroke CRPS, which aids in the identification of patients who are at high risk. A previous study found a 2.17 fold of increase in the healthcare utilization cost after diagnosis of CRPS in the general population, and such increase persisted at least 8 years after diagnosis [45]. Furthermore, oral corticosteroids are currently the only anti-inflammatory drugs with level 1 evidence [2]. However, this treatment remains problematic for its adverse events, including hyperglycemia and hypertension [46], which were common comorbid diseases in stroke patients [47]. Hence, prevention methods should be applied after stroke, especially in those with high risk of developing poststroke CRPS. Calcitonin, early rehabilitation, and restriction of passive movement of the affected limb have been examined through clinical trials for prevention of poststroke CRPS [6,7,48]. Starting early rehabilitation not only for the prevention of CRPS but also shoulder subluxation in the acute phase with the aid of adequate nursing care is essential [49]. Rehabilitation centers may utilize the findings in our study to increase the outcomes of poststroke patients.

## 5. Conclusions

This article of meta-analysis and meta-regression revealed that being female, left hemiparesis, shoulder subluxation, spasticity, a lower Brunnstrom hand stage, and inferior Barthel index are risk factors for development of poststroke CRPS. Larger studies with greater power may confirm our findings and clarify some other unknown risk factors for poststroke CRPS.

## Figures and Tables

**Table 1:** Characteristics of all included studies.

Research	Country/Area, Year of Study	Study Design	Study Population	Study Size	Age (Years)	Sex (% of Females)
Lee et al., 2021 [8]	November 2012 to August 2019, Korea	Retrospective nested case-control study	Hospitalized, first-ever episode of ischemic stroke, unilateral hemispheric, supratentorial	CRPS group: 35, control group: 110	CRPS group: median 72 (IQR 65–78); control group: median 72 (IQR 58.75–80))	42.1%
Kim et al., 2020 [4]	January 2009 and May 2019, Korea	Cross-sectional study	Hospitalized, first-ever episode of stroke within 3 months, supratentorial	CRPS group: 38, control group: 42	CRPS group: 67.9 (10.3); control group: 62.7 (10.9)	43.8%
Altas et al., 2020 [20]	June 1, 2014 and June 1, 2019, Turkey	Retrospective nested case-control study	Hospitalized, stroke	CRPS group: 213, control: 213	CRPS group: 67.9 (10.3); control group: 66.1 (9.9)	60.1%
Oh et al., 2019 [21]	May 2016 and June 2017, Korea	Cross-sectional study	Hospitalized, first-ever stroke with hemiplegia	CRPS group: 66, control group: 6	CRPS group: 50.8 (SEM 5.7); control group: 48.9 (SEM 10.9)	22.2%
Yetisgin et al., 2017 [22]	January 2015 to May 2016, Turkey	Cross-sectional study	Outpatient clinic, first ever stroke with hemiplegia	CRPS group: 10, control group: 43	CRPS group: 60.6 (16.2); control group: 59.8 (12.7)	41.5%
Han et al., 2014 [23]	January, 2003, to December, 2007, Korea	Retrospective nested case-control study	Hospitalized, first ever stroke, and performed somatosensory evoked tests of potentials in the hemiparetic limb 2–4 weeks post stroke	CRPS group: 70, control group: 182	CRPS group: 61.5 (12.8); control group: 63.1 (13)	43.7%
Kitisomprayoonkul et al., 2010 [24]	August 2006 to January 2007, Thailand	Prospective nested case-control study	Hospitalized for stroke	118	63.4 (11.2)	39.0%
Barlak et al., 2009 [25]	January 2005 and May 2007, Turkey	Cross-sectional study	Hospitalized, first ever unilateral stroke	CRPS group: 114, control group: 73	61.3 (11.3)	50.8%
Kocabas et al., 2007 [26]	September 2002 to May 2003, Turkey	Prospective nested case-control study	Hospitalized, stroke	CRPS group: 40, control group: 42	64.0 (9.9)	53.7%
Selçuk et al., 2006 [27]	2000 to 2005, Turkey	Cross-sectional study	Stroke with hemiplegia or hemiparesis	CRPS group: 41, control (stroke) group: 28, control (healthy): 20	Stroke patients: 60.0 (12.9); healthy individuals: 57.9 (15.6)	37.7%
Gokkaya et al., 2006 [28]	NR, Turkey	Cross-sectional study	Hospitalized for stroke	CRPS group: 29, control group: 66	CRPS group: 60.7 (11.4); control group: 58.7 (12)	33.7%
Aras et al., 2004 [29]	NR, Turkey	Cross-sectional study	Hospitalized for stroke	85	59.5 (11.7)	30.6%
Iwata et al., 2002 [30]	NR, Japan	Prospective nested case-control study	Hospitalized, first ever unilateral stroke within 3 weeks	CRPS group: 8, control group: 26	CRPS group: 58.9 (8.7); control group: 62.5 (9.7)	41.2%
Daviet et al., 2002 [31]	November 1997 to August 1998, France	Prospective cohort study	Hospitalized for stroke with hemiplegia within 1 month	69	NR	42.0%
Dachy et al., 2002 [32]	NR, Belgium	Prospective cohort study	Hospitalized for first ever hemispheric stroke	20	67.9 (range 41–85)	45%
Dursun et al., 2000 [33]	NR, Turkey	Cross-sectional study	Hospitalized for first ever unilateral stroke with Ashworth scale 2 and below	CRPS group: 35, control group: 35	CRPS group: 59.1 (SEM 1.6); control group: 59.8 (SEM 1.7)	45.7%
Hesse et al., 1995 [34]	NR, Germany	Cross-sectional study	Stroke with hemiparesis	CRPS group: 21, control group: 18	60.6 (range 43–79)	33.3%
Cheng et al., 1995 [35]	NR, Taiwan	Prospective nested case-control study	Hospitalized for first ever stroke within 3 weeks	CRPS group: 31, control group: 39	62.3	42.9%
Braus et al., 1994 [36]	NR, Germany	Prospective nested case-control study	Hospitalized for stroke with hemiplegia	CRPS group: 36, control group: 96	61.6 (11.9)	31.8%
Weiss et al., 1993 [37]	NR, United States	Prospective cohort study	Hospitalized for stroke with hemiparesis	22	NR	50.0%
Tepperman et al., 1984 [38]	NR, Canada	Cross-sectional study	Hospitalized for stroke with hemiplegia	85	CRPS group: 66.6 (8.3); control group: 66.7 (10.4)	43.5%

Results are given as mean (standard deviation), unless otherwise noted; CRPS: complex regional pain syndrome; SEM: standard error of mean.

**Table 2:** Summary of extracted data from the included studies.

Research	Time of Outcome Assessment	% Developing CRPS (CRPS/Total)	Criteria	Risk Factors Assessed with Statistical Significance	Risk Factors Assessed without Statistical Significance
Lee et al., 2021 [8]	NR	24.1% (35/145)	Budapest Criteria	Manual Function Test, Modified Barthel Index, Fugl‒Meyer assessment (total and upper extremity), strength of shoulder flexion and wrist extension of the hemiplegic side (MRC), Berg Balance Scale, MMSE, somatosensory evoked potentials in median nerve, damage to the white matter of the CST, caudate nucleus, and putamen	Age and sex, presence of shoulder subluxation and spasticity, stroke lesion location (middle cerebral arterial region or others), and lesion volume
Kim et al., 2020 [4]	NR	47.5% (38/80)	Budapest criteria	pain intensity of affected wrist, spasticity (Modified Ashworth Scale), strength of shoulder (MRC), medication during admission (Medication Quantification Scale) scores, shoulder subluxation; damage to the white matter of the CST	Age, sex, etiology of stroke, side of lesion, duration of stroke, lesion volume, MMSE, Geriatric Depression Scale, Modified Barthel Index, Prevalence of shoulder pain at rest
Altas et al., 2020 [20]	NR	NR	Budapest criteria	Duration of stroke, time to hospitalization, admission duration, coronary artery disease, upper extremity Brunnstrom stage, Brunnstrom hand stage, spasticity (Modified Ashworth scale), Barthel index, shoulder subluxation, shoulder soft tissue lesion, adhesive capsulitis, previous orthopedic surgeries, fracture in the upper extremities, neglect, visual field defect, heterotopic ossification, entrapment neuropathy, pressure wound, lower respiratory tract infection, urinary infection, epilepsy, depression	Age, sex, affected side, etiology of stroke, smoking, hypertension, diabetes, atrial fibrillation, dyslipidemia, deep vein thrombosis, brachial plexus injury, protein-energy malnutrition
Oh et al., 2019 [21]	NR	8.3% (6/72)	Budapest criteria	Etiology of stroke	Age, sex, affected side, disease duration, strength of elbow, wrist, shoulder summarized (MRC), Brunnstrom stage, spasticity (Modified Ashworth scale), shoulder subluxation (acromion-greater tuberosity distance)
Yetisgin et al., 2017 [22]	NR	18.9% (10/53)	NR	Sex, Brunnstrom hand stage, shoulder pain	Age, etiology of stroke, affected side, duration after stroke, rehabilitated, Brunnstrom stage of arm and lower extremity, functional ambulation scale
Han et al., 2014 [23]	NR	27% (70/252)	Orlando criteria	Etiology of stroke, somatosensory evoked potentials in median nerve, shoulder subluxation	Age, sex, stroke lesion location (cortical or subcortical), affected side
Kitisomprayoonkul et al., 2010 [24]	Until discharge from hospital (mean 122.5 days)	15.3% (18/118)	Bonica’s management of pain. 3rd edition	Limit range of motion of weak shoulder	NR
Barlak et al., 2009 [25]	NR	37.4% (70/187)	Bonica’s management of pain. 3rd edition	Brunnstrom stage	Shoulder subluxation
Kocabas et al., 2007 [26]	28 weeks after stroke	48.8% (40/82)	Bonica’s management of pain. 3rd edition	Spasticity (Ashworth scale), Brunnstrom score, strength of arm (Motricity Index), shoulder ROM, shoulder subluxation, depression (regression analysis of Beck score)	Sex, affected side, etiology of stroke, presence of hypoesthesia, leg strength (Motricity Index), Barthel index, depression (mean difference in Beck score)
Selçuk et al., 2006 [27]	NR	59.4% (41/69)	Kozin’s clinical criteria	Sympathetic skin responses (absent or not, amplitude)	Sympathetic skin responses (latency)
Gokkaya et al., 2006 [28]	NR	30.5% (29/95)	Clinical criteria of Tepperman et al., 1984	Brunnstrom stage, shoulder subluxation	Age, sex, affected side, etiology of stroke, disease duration, duration of admission for rehabilitation
Aras et al., 2004 [29]	NR	30.6% (26/85)	Clinical criteria of Tepperman et al., 1984	Shoulder subluxation	NR
Iwata et al., 2002 [30]	2–4 months after stroke	23.5% (8/34)	Clinical diagnosis	Ratio circumference of the middle finger between hands	Age, sex, affected side, etiology of stroke, hemispatial neglect, dominant hand, Brunnstrom arm and hand stage
Daviet et al., 2002 [31]	3 months after stroke	34.8% (24/69)	Labrousse severity scale	Severity of symptoms (Labrousse scale), strength (Motricity Index), spasticity (Ashworth scale), shoulder subluxation, length of stay in acute ward, initial coma, Perrigot score	Age, sex, affected side, vibration sensitivity, depression (Montgomery-Asberg Depression Rating Scale), hemineglect, proprioception, diabetes, hypertension, dyslipidemia, thyroid disorder, barbituric treatment, anticoagulant treatment, edema of hand and forearm
Dachy et al., 2002 [32]	70 days after stroke	NR	Enjalbert score	Transcranial magnetic stimulation induced motor evoked potential	NR
Dursun et al., 2000 [33]	NR	50% (35/70)	Either clinical or bone scintigraphy	Shoulder subluxation	Age, sex, side, etiology of stroke, duration of disease, Brunnstrom stage
Hesse et al., 1995 [34]	NR	NR	Clinical diagnosis	Amplitude, area, F/M ratio of sympathetic skin response, temperature difference between limbs	NR
Cheng et al., 1995 [35]	6 months after admission	44.2% (31/70, including 15 definite, 9 probable and 7 possible)	Clinical criteria of Tepperman et al., 1984	Sensory impairment, shoulder subluxation, spontaneous electromyography activity of affected limb,	Age, sex, spasticity, etiology of stroke, affected side, distal latency and amplitude of thenar compound muscle action potential
Braus et al., 1994 [36]	6 months after discharge	27.2% (36/132, all definite)	Kozin et al., 1981	Subluxation, motor deficit, spasticity (Ashworth scale), deficits in confrontation visual field testing	Age, sex, affected side, etiology of stroke
Weiss et al., 1993 [37]	6 months after admission	54.5% (12/22)	Clinical diagnosis	NR	Sex, sensory deficit, side
Tepperman et al., 1984 [38]	NR	24.7% (21/85)	Bone scintigraphy	Peptic ulcer, vasomotor changes, wrist tenderness, metacarpal phalangeal joint tenderness, interphalangeal joint tenderness	Age, sex, affected side, etiology of stroke, hypertension, diabetes mellitus, atherosclerotic heart disease, congestive heart failure, atrial fibrillation, peripheral vascular disease, chronic obstructive lung disease, mitral stenosis, shoulder pain and tenderness, swelling of the wrist and hand

CST: corticospinal tract; MMSE: Mini-Mental State Examination; MRC: Medical Research Council’s scale; NR: not reported; ROM: range of motion.

**Table 3:** Risk of bias assessment for cohort and case-control studies.

Newcastle-Ottawa Scale	Selection				Comparability		Outcome			Total Score
Author, Year	1	2	3	4	5	6	7	8	9	
^a^ Cohort										
Weiss et al., 1993 [37]	*	*	*	-	-	-	*	*	*	6
Daviet et al., 2002 [31]	*	*	*	-	-	-	*	*	*	6
Dachy et al., 2002 [32]	*	*	*	*	-	-	*	*	*	7
^b^ Case-control										
Braus et al., 1994 [36]	*	*	*	*	-	-	*	*	*	7
Cheng et al., 1995 [35]	*	*	*	*	-	-	*	*	*	7
Iwata et al., 2002 [30]	*	-	*	*	-	-	*	*	*	6
Kocabas et al., 2007 [26]	*	*	*	*	-	-	*	*	*	7
Kitisomprayoonkul et al., 2010 [24]	*	*	*	*	-	-	*	*	*	7
Han et al., 2014 [23]	*	*	*	*	-	-	*	*	*	7
Altas et al., 2020 [20]	*	*	*	*	-	-	*	*	*	7
Lee et al., 2021 [8]	*	*	*	*	-	-	*	*	*	7

Studies were assessed for risk of bias by the Newcastle–Ottawa Scale for cohort or case control studies. *: The criteria were met; -: The criteria were not met. ^a^ 1: Representativeness of the exposed cohort; 2: Selection of the non-exposed cohort; 3: Ascertainment of exposure; 4: Demonstration that outcome of interest was not present at start of study; 5: Comparability of cohorts on the basis of the design or analysis; 6: Comparability of cohorts on the basis of the design or analysis, controls for any additional factor; 7: Assessment of outcome; 8: Was follow-up long enough for outcomes to occur; 9: Adequate of follow-up of cohorts. ^b^ 1: Is the case definition adequate; 2: Representativeness of the cases; 3: Selection of controls; 4: Definition of controls; 5: Comparability of cases and controls on the basis of the design or analysis; 6: Comparability, controls for any additional factor; 7: Ascertainment of exposure; 8: Same method of ascertainment for cases and controls, 9: Non response rate.

## Data Availability

No new data were created in this study.

## Supplementary Materials

The following are available online at https://www.mdpi.com/article/10.3390/medicina57111232/s1, File S1: Search strategies for meta-analysis, File S2. Funnel plots for publication bias and bubble plots of meta-regression, File S3. Details of articles excluded after full text retrieved.
