# Supplementary material for: A Meta-Analysis and Meta-Regression of Frequency and Risk Factors for Poststroke Complex Regional Pain Syndrome
Source: Medicina (Kaunas). 2021 Nov 11;57(11):1232. doi: 10.3390/medicina57111232 (PMC8622266; doi:10.3390/medicina57111232)
Supplement: Supplementary file 1 [file medicina-57-01232-s001.zip › File S1.pdf]

File S1. Search strategies for meta-analysis

The search was performed on October 27, 2021.

PUBMED Results: 223

#16 Search: #8 AND #14 AND English[filter] 223

#15 Search: #8 AND #14 289

#14 Search: #9 OR #10 OR #11 OR #12 OR #13 503100

#13 Search: cerebrovascular 150324

#12 Search: hemipare* 12844

#11 Search: hemiple* 18220

#10 Search: stroke 384773

#9 Search: stroke[Mesh] 150717

#8 Search: #1 OR #2 OR #3 OR #4 OR #5 OR #6 OR #7 7703

#7 Search: “reflex sympathetic dystrophy” 4167

#6 Search: sudek* 184

#5 Search: Algodystroph* 560

#4 Search: "shoulder-hand syndrome" 341

#3 Search: "shoulder hand syndrome" 341

#2 Search: "complex regional pain syndrome" 3250

#1 Search: "complex regional pain syndromes"[MeSH Terms] 5751

Medline Results: 222

S16 S8 AND S14 Limiters - English Language 222

S15 S8 AND S14 288

S14 S9 OR S10 OR S11 OR S12 OR S13 490890

S13 cerebrovascular 149438

S12 hemipare* 12772

S11 hemiple* 18178

S10 stroke 366948

S9 mh(stroke+) 150004

S8 S1 OR S2 OR S3 OR S4 OR S5 OR S6 OR S7 7674

S7 “reflex sympathetic dystrophy” 4160

S6 sudek* 183

S5 Algodystroph* 548

S4 "shoulder-hand syndrome" 3039

S3 "shoulder hand syndrome" 3039

S2 “complex regional pain syndrome” 3447

S1 MH(complex regional pain syndromes+) 5741

Cochrane Central Register of Controlled Trials Results: 121

#16 #7 AND #13 in trials 121

#15 #7 AND #13 159

#14 #9 OR #10 OR #11 OR #12 OR #13 82625

#13 cerebrovascular 24865

#12 hemipare* 2222

#11 hemiple* 3166

#10 stroke 74131

#9 MeSH descriptor: [Stroke] explode all trees 10731

#8 #1 OR #2 OR #3 OR #4 OR #5 OR #6 OR #7 894

#7 “reflex sympathetic dystrophy” 327

#6 sudek* 4

#5 Algodystroph* 50

#4"shoulder-hand syndrome" 95

#3 "shoulder hand syndrome" 95

#2 "complex regional pain syndrome" 645

#1 MeSH descriptor: [Complex Regional Pain Syndromes] explode all trees 312

Web of Science Results: 376

#14 #7 AND #12 AND LA=(English) 376

#13 #7 AND #12 392

#12 #8 OR #9 OR #10 OR #11 704025

#11 ALL=(cerebrovascular) 80128

#10 ALL=(hemipare*) 11692

#9 ALL=(hemiple*) 13527

#8 ALL=(stroke) 652117

#7 #1 OR #2 OR #3 OR #4 OR #5 OR #6 7189

#6 ALL=("reflex sympathetic dystrophy") 2912

#5 ALL=(sudek*) 416

#4 ALL=(Algodystroph*) 469

#3 ALL=(shoulder-hand syndrome) 216

#2 ALL=(shoulder hand syndrome) 1490

#1 ALL=(complex regional pain syndrome) 3471

Embase Results: 569

#14 #7 AND #12 AND [English]/lim 569

#13 #7 AND #12 685

#12 #8 OR #9 OR #10 OR #11 720257

#11 cerebrovascular 473848

#10 hemipare* 31372

#9 hemiple* 27085

#8 stroke 505696

#7 #1 OR #2 OR #3 OR #4 OR #5 OR #6 14423

#6 reflex sympathetic dystrophy 2548

#5 sudek* 192

#4 Algodystroph* 1238

#3 shoulder-hand syndrome 483

#2 shoulder hand syndrome 2814

#1 complex regional pain syndrome 10008
