# Supplementary material for: A Meta-Analysis and Meta-Regression of Frequency and Risk Factors for Poststroke Complex Regional Pain Syndrome
Source: Medicina (Kaunas). 2021 Nov 11;57(11):1232. doi: 10.3390/medicina57111232 (PMC8622266; doi:10.3390/medicina57111232)
Supplement: Supplementary file 1 [file medicina-57-01232-s001.zip › File S2.pdf]

File S2. Funnel plots for publication bias and bubble plots of meta-regression

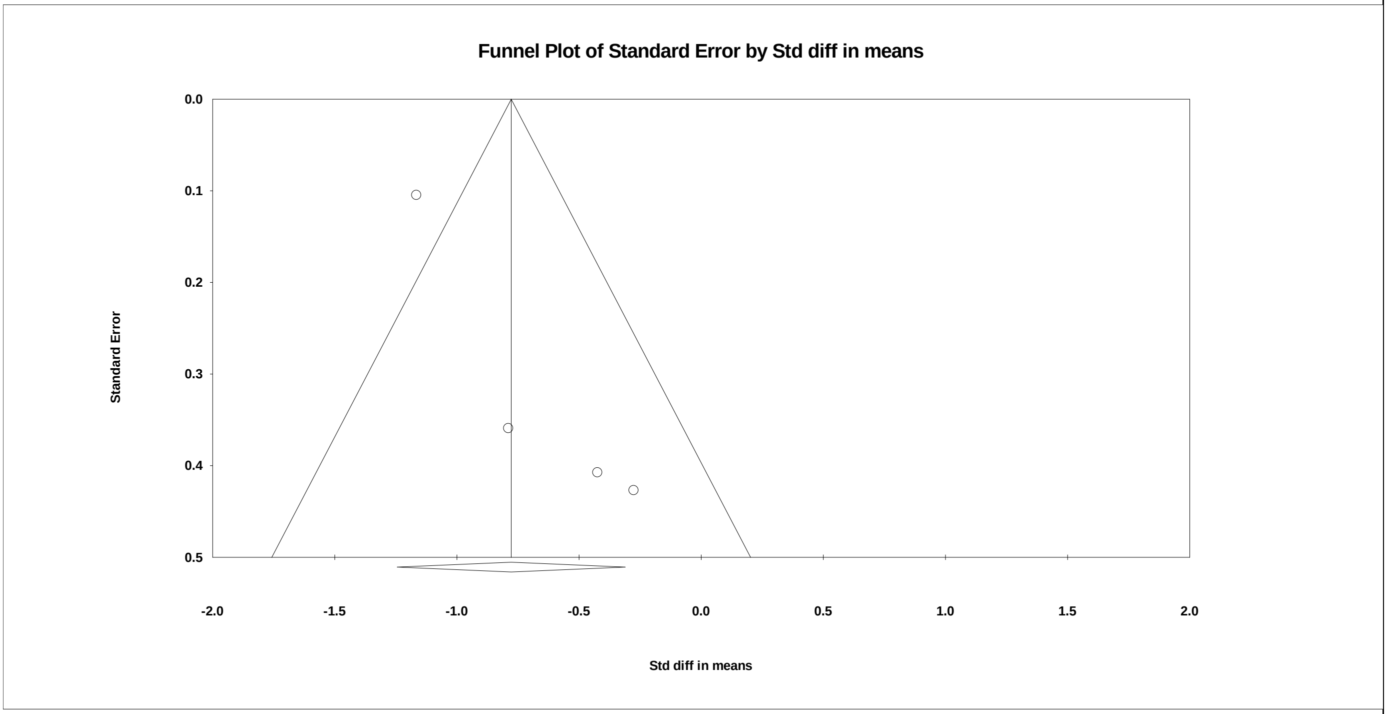

File S2. (a) Funnel plot of Brunnstrom hand stage

Significant publication bias was detected when assessing the Brunnstrom hand stage as a possible risk factor of poststroke complex regional pain syndrome (CPRS).

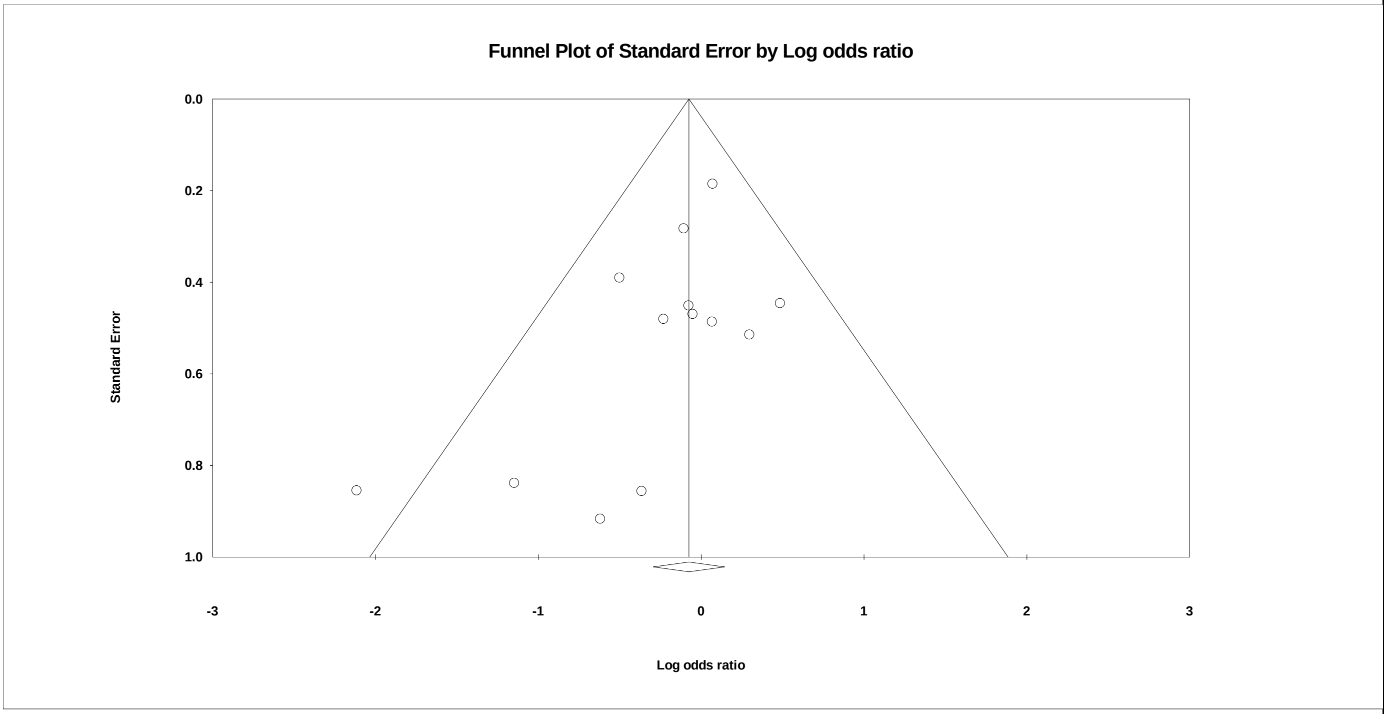

File S2. (b) Funnel plot of sex

Significant publication bias was detected when assessing sex as a possible risk factor of poststroke CRPS.

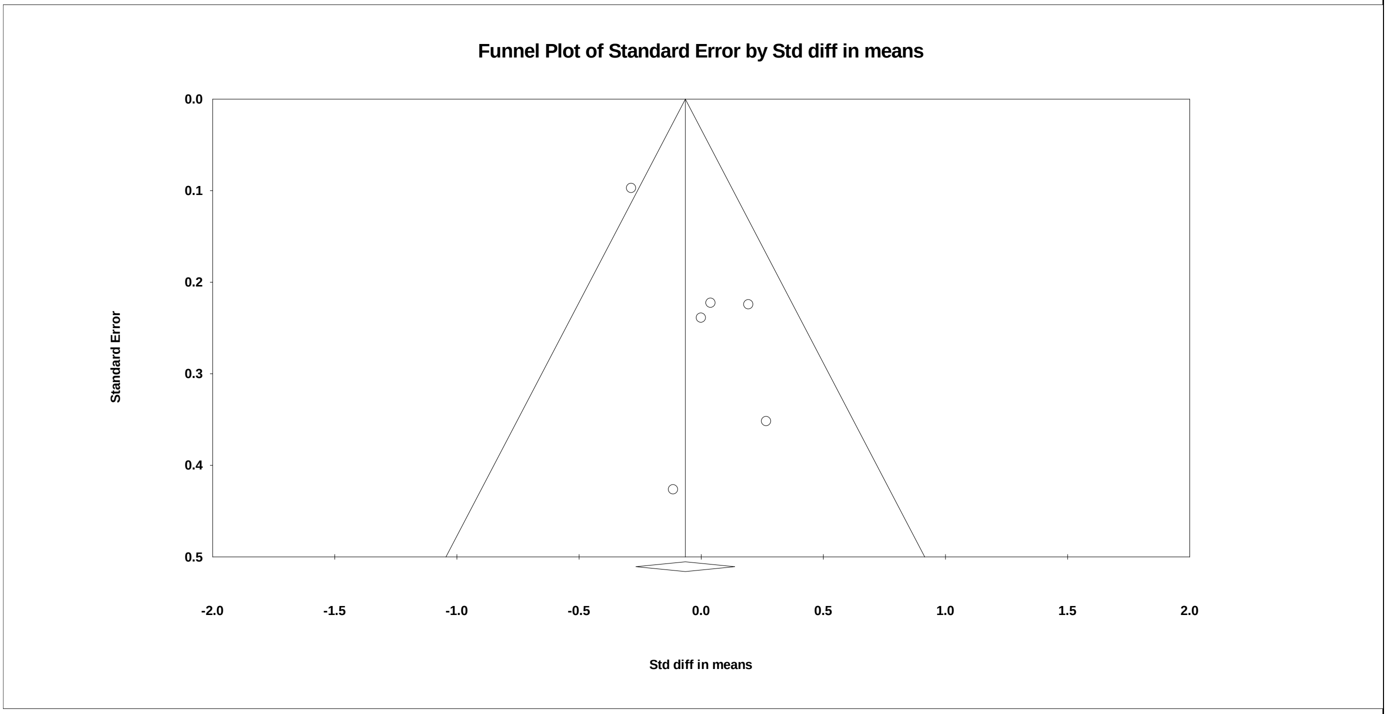

File S2. (c) Funnel plot of duration of stroke

Significant publication bias was detected when assessing duration of stroke as a possible risk factor of poststroke CRPS.

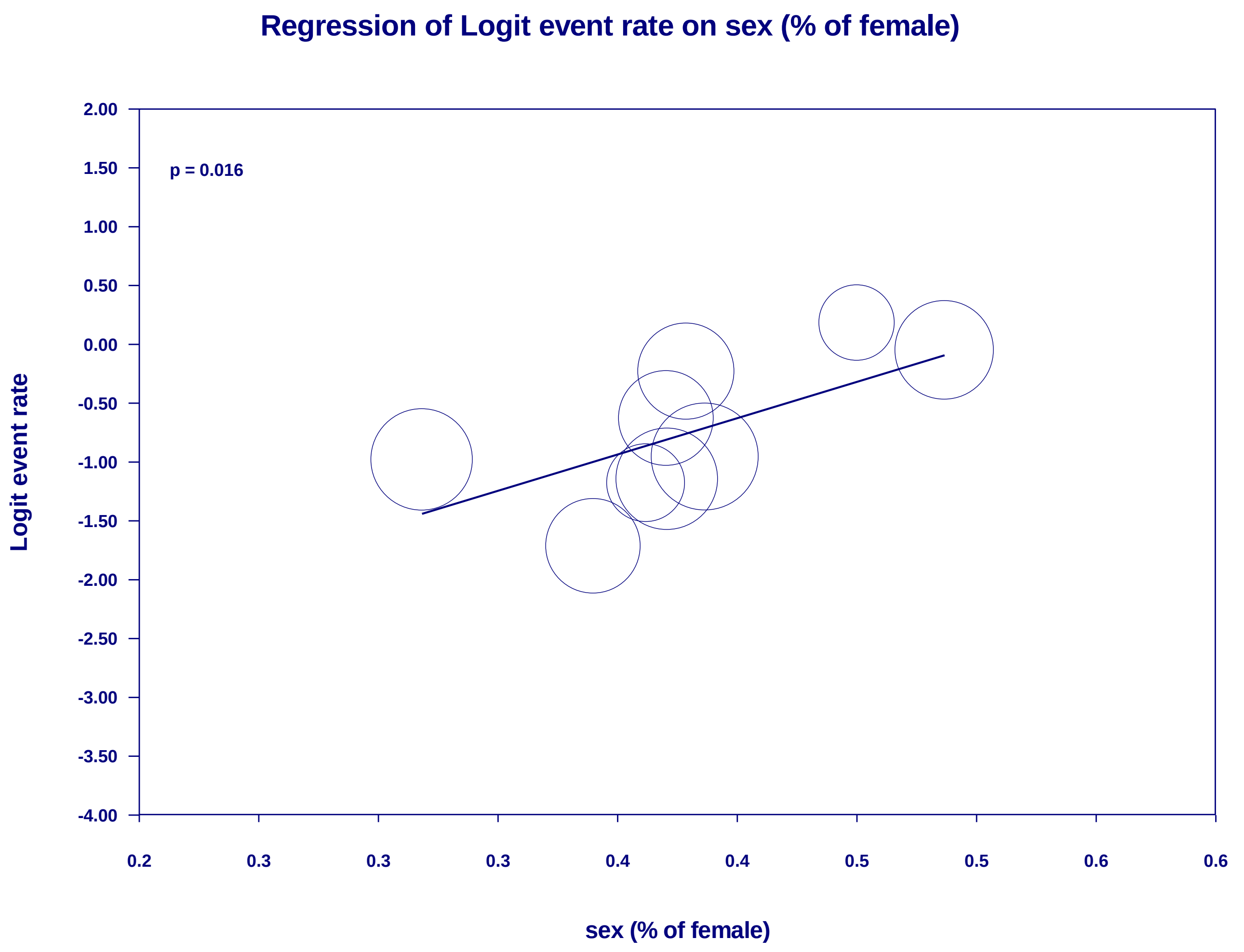

File S2. (d) Bubble plot of meta-regression between sex and incidence proportion

A positive correlation between the proportion of female in the studies and the incidence proportion

of poststroke CRPS was revealed. Size of the circles indicated the weight of each included study.

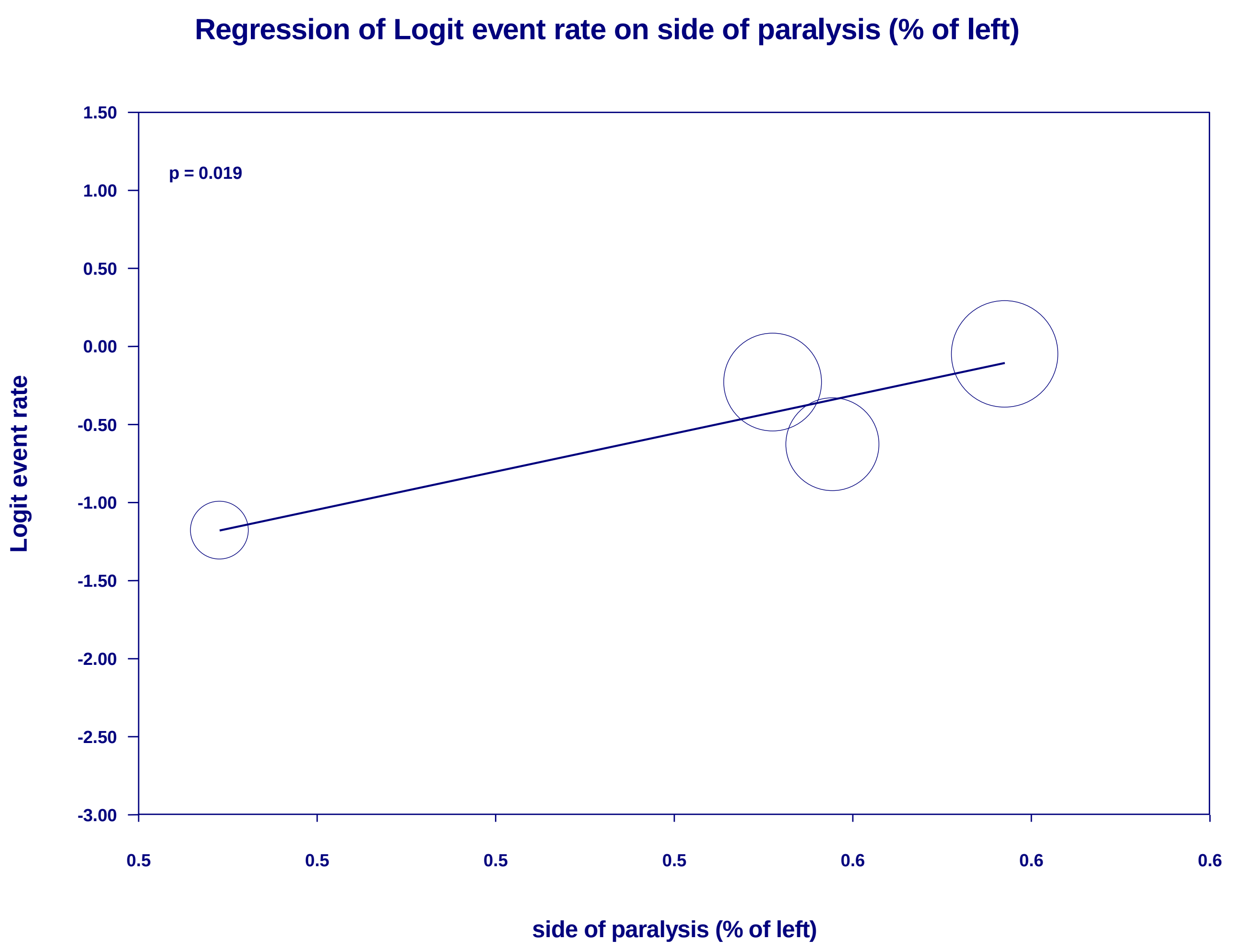

File S2. (e) Bubble plot of meta-regression between side of paralysis and incidence proportion

A positive correlation between the proportion of left side paralysis in the studies and the incidence proportion of poststroke CRPS was revealed. Size of the circles indicated the weight of each included study.
