# Supplementary material for: A Meta-Analysis and Meta-Regression of Frequency and Risk Factors for Poststroke Complex Regional Pain Syndrome
Source: Medicina (Kaunas). 2021 Nov 11;57(11):1232. doi: 10.3390/medicina57111232 (PMC8622266; doi:10.3390/medicina57111232)
Supplement: Supplementary file 1 [file medicina-57-01232-s001.zip › File S3.pdf]

File S3. Articles excluded after full text retrieved

1	Park et al., Use of Oral Prednisolone and a 3-Phase Bone Scintigraphy in Patients with Complex Regional Pain Syndrome Type I. Healthcare (Basel). 2020 Jan 9;8(1):16.	mixing other etiology of hemiplegia other than stroke
2	Park et al., Patterns of three-phase bone scintigraphy according to the time course of complex regional pain syndrome type I after a stroke or traumatic brain injury. Clin Nucl Med. 2009 Nov;34(11):773-6.	mixing other etiology of hemiplegia other than stroke
3	Kumar et al., A study of bone densitometry in patients with complex regional pain syndrome after stroke. Postgrad Med J. 2001 Aug;77(910):519-22.	examining risk factors within a general population sample
4	Choi et al., Relationship Between HbA1c and Complex Regional Pain Syndrome in Stroke Patients With Type 2 Diabetes Mellitus. Ann Rehabil Med. 2016 Oct;40(5):779-785.	restricted to specific clinical subgroups of patients with stroke (diabetes mellitus)
5	Wang et al., Reflex sympathetic dystrophy syndrome in stroke patients with hemiplegia-three phase bone scintigraphy and clinical characteristics. Kaohsiung J Med Sci. 1998 Jan;14(1):40-7.	restricted to specific clinical subgroups of patients with stroke (limb discomfort)
6	Lo et al., Arthrographic and clinical findings in patients with hemiplegic shoulder pain., Arch Phys Med Rehabil. 2003 Dec;84(12):1786-91.	restricted to specific clinical subgroups of patients with stroke (hemiplegic shoulder pain)
7	Liu et al., Effect of rehabilitation therapy and nursing intervention on postoperative recovery of patients with hypertensive intracerebral hemorrhage. Experimental and Therapeutic Medicine 2019 Jun;17(6):4598-4604	restricted to specific clinical subgroups of patients with stroke (intracranial hemorrhage needing operation)
8	Greyson et al., Three-phase bone studies in hemiplegia with reflex sympathetic dystrophy and the effect of disuse. J Nucl Med. 1984 Apr;25(4):423-9.	Only assessing bone scan as a determinant
9	Park et al., Quantitative evaluation of very acute stage of complex regional pain syndrome after stroke using three-phase bone scintigraphy. Nucl Med Commun. 2007 Oct;28(10):766-70	Only assessing bone scan as a determinant
10	Barlak et al., Poststroke shoulder pain in Turkish stroke patients: relationship with clinical factors and functional outcomes. Int J Rehabil Res. 2009 Dec;32(4):309-15.	Excluded during quantitative synthesis (No quantitative data available; no respond from authors by email)
